# Myofascial trigger points alter the modular control during the execution of a reaching task: a pilot study

**DOI:** 10.1038/s41598-019-52561-3

**Published:** 2019-11-05

**Authors:** Tommaso Geri, Leonardo Gizzi, Anna Di Marco, Marco Testa

**Affiliations:** 1 Department of Neuroscience, Rehabilitation, Ophthalmology, Genetics, Maternal and Child Health (DINOGMI), University of Genova, Campus of Savona, Genova, Italy; 2 Institute for Modelling and Simulation of Biomechanical Systems, Continuum Biomechanics and Mechanobiology Research Group, University of Stuttgart, Stuttgart, Germany

**Keywords:** Spinal cord, Skeletal muscle, Biomedical engineering

## Abstract

Myofascial trigger points (TP) constitute a conundrum in research and clinical practice as their etiopathogenesis is debated. Several studies investigating one or few muscles have shown that both active and latent TP causes an increased muscle activity, however the influence of TP on modular motor control during a reaching task is still unclear. Electromyographic signals, recorded from the muscles of the shoulder girdle and upper arm during a reaching task, were decomposed with Non-Negative Matrix Factorization algorithm. The extracted matrices of motor modules and activation signals were used to label the muscles condition as dominant or non-dominant. The presence of latent and active TP was detected in each muscle with manual examination. Despite a similar muscle activity was observed, we found that muscles with active TP had increased weighting coefficients when labeled in the dominant condition. No influences were found when muscles were in the non-dominant condition. These findings suggest that TP altered the motor control without co-contraction patterns. As a preliminary evidence, the present results suggest that the increased weighting coefficients in presence of TPs are associated with an alteration of the modular motor control without affecting the dimensionality of motor modules for each individual and reciprocal inhibition.

## Introduction

Trigger Points (TPs) are clinical entities commonly found in several musculoskeletal conditions as well as in healthy subjects^1^. A TP is defined as a “hyperirritable spot in skeletal muscle that is associated with a hypersensitive palpable nodule in a taut band”^2^. It can have two states according to the reproduction of the patient’s current or past symptoms (active TP) or not (latent TP) upon palpation^3^. Other features, such as a tender spot within a taut band, referred pain, and the presence of a local twitch response are common to the two states. Some explanations have been advanced to describe the development of TPs and their role on musculoskeletal pain^4–11^, however there is debate on the origin of the primary nociceptive input causing the sensitization of the CNS (Central Nervous System) that leads to spot tenderness and referred pain^12,13^.

The characteristic motor signs of a TP are the taut band and the local twitch response. The taut band is likely due to the Spontaneous Electrical Activity (SEA) observed at rest at the site of TP^14^, which is considered as extrafusal or intrafusal according to the withstanding hypotheses. The integrated trigger point hypothesis considers an extrafusal origin of the SEA as the combination of miniature endplate potentials and endplate spikes that cause an abnormal acetylcholine release due to muscle damage occurring in extraordinary activities or following a trauma^15^. On the other hand, it has also been suggested that SEA may be the electrical activity of muscle spindles which constitute the nociceptive locus from which TP and myofascial pain begins^6^. The local twitch response is commonly seen as an augmented arch reflex response, intended as stretch^4,5^ or withdrawal reflex^7^.

Motor alterations were found also for latent TPs^16–18^ and characterized both as “within-” and “between-muscles”. Within-muscle alterations comprise higher muscle spindle sensitivity, with higher amplitude and lower threshold of the H-reflex^19^, and increased metabolic fatigability^18^. Between-muscles alterations have been reported as either an increase in the activity of antagonist^20^ as well as synergistic muscles^17^ or as a delayed activation with respect to the other agonist muscles^21^ of the muscle containing the TP. These phenomena are also associated with a heterogeneous redistribution of activity of the other muscles during, for example, either slow^21^ or rapid^22^ arm elevation. This body of evidences may suggest that the nociceptive afferents from a TP induce alterations of the spinal circuitry whose efferent pathway causes a focal dystonia of the muscle involved^10^.

The CNS accomplishes the problem of controlling its numerous degrees of freedom during the continuous interaction with the environment by controlling a small set of elemental variables, also called motor modules^23^. A motor module is a group of muscles controlled as a single unit^24^ that is spatially and temporally characterized through the directional tuning of its weighting and timing coefficients, respectively^25^. This approach has already been used to describe complex human motor behaviors like walking, in infants^26^, healthy adults^27^ and neurological patients^28^; reaching task, in healthy^29–31^ and stroke subjects^32^; and in presence of various musculoskeletal pain syndromes^33^. Despite an influence of TP on motor modules has been hypothesized^17^, an investigation of the influence of TP at the level of modular motor control has not been performed so far.

In this study we aimed at exploring the role of active and latent TPs on the modular control of reaching movements. Upper limb muscular activities from the shoulder girdle and arm muscles were analyzed by means of Non-negative Matrix Factorization (NMF)^34^ and dimensionality of control, while weighting and timing coefficients were extracted. The results were interpreted in light of the presence or absence of TPs. We hypothesized that the presence and number of TP may alter the individual dimensionality of the motor modules (Hypothesis 1). Furthermore, we expected that the presence of TPs altered the weighting coefficients of motor modules, possibly influencing the weighting coefficient of the hosting muscle in each synergy (Hypothesis 2). Moreover, we investigated whether muscle activity was also altered across muscles with or without TPs (Hypothesis 3). Finally, we generated a random presentation of TPs among the investigated muscles to understand whether our results might be influenced by a spurious association due to flaws in the palpation-based diagnosis of TPs (Hypothesis 4).

## Results

### Subjects

The 15 participants (7 F) had an average age of 28.13 ± 4.02 years. Six participants (3,7,9,10,12,14) reported musculoskeletal pain of the upper quarter, subject 8 a history of headaches and subject 13 of low back pain. The remaining 7 subjects had no history of musculoskeletal pain. The remaining demographic variables and scores of self-reported questionnaires are reported in Table 1.

**Table 1:** Demographic variables and self-reported questionnaire scores.

Variable	N	Mean (SD)	Min	Max
Age (years)	15	28.13 (4.02)	22	34
Weight (Kg)	15	66.33 (11.18)	51	90
Height (cm)	15	171.2 (8.28)	160	186
VAS (0–10)	15	1.65 (1.88)	0	5.90
EQI (0–1)	15	0.89 (0.09)	0.76	1
NBQ – Functioning subscale (0–40)	15	3.33 (3.74)	0	10
NBQ – Anxiety subscale (0–20)	15	2.86 (3.38)	0	10
QuickDASH (11–55)	15	11.93 (1.16)	11	14
TSK (13–52)	15	16.47 (5.08)	13	31
PCS (0–52)	15	3.67 (3.87)	0	11

EQI, EuroQol Index, range from 0 (worst) to 1 (best); NBQ, Neck Bournemouth Questionnaire, subscales range from 0 (best) to 20/40 (worst); PCS, Pain Catastrophizing Scale, range from 0 (best) to 52 (worst); QuickDASH, Quick Disability of the Arm, Shoulder and Hand questionnaire, range from 11 (best) to 55 (worst); SD, Standard Deviation; TSK, Tampa Scale for Kinesiophobia, range from 13 (best) to 52 (worst); VAS, Visual Analogue Scale for pain intensity, range from 0 (best) to 10 (worst).

### Trigger points detection

The presence of TP is reported for subjects and muscles in Table 2. Subject 4 had no TPs detected in the muscles examined, two subjects had only one active TP each, while seven subjects had only latent TPs. The remaining 5 subjects had both active and latent trigger points (Table 2). The muscles with the highest number of both active and latent TPs were the SCOM (4 ACT, 5 LAT) and the TLO (2 ACT, 7 LAT). The muscles with active and latent TPs were, respectively, 13 (6%) and 50 (25%) on a total of 195 examined muscles.

**Table 2:** Result of muscle palpation for TP detection.

Subject	VAS	ACT (muscle)	LAT (muscle)
01	0	0	3 (BS, TM, SCOM)
02	0	0	4 (BR, TM, TLO, TU)
03	3.8	1 (DA)	1 (TLO)
04	0	0	0
05	0	0	2 (PM, TU)
06	0	0	2 (DP, PM)
07	5.9	1 (TM)	5 (BS, PM, SCOM, TLO, TU)
08	3	0	8 (BS, BR, DP, TM, PM, SCOM, TLO, TU)
09	3.7	0	8 (BS, BR, DM, DP, PM, SCOM, TLA, TLO)
10	1.5	1 (SCOM)	5 (DP, PM, TLA, TLO, TU)
11	0	1 (SCOM)	0
12	2	1 (SCOM)	0
13	2	4 (BR, PM, TLA, TLO)	4 (BL, BS, DA, SCOM)
14	2.8	4 (DM, TM, SCOM, TLO)	4 (BS, TL, PM, TU)
15	0	0	4 (BL, BS, TM, TLO)

ACT, Active trigger point; BL, Biceps Long head; BS, Biceps Short head; BR, Brachioradialis; DA, Deltoid Anterior; DM, Deltoid Middle; DP, Deltoid Posterior; LAT, Latent trigger point; TL, Trapezius Lower; TM, Trapezius Middle; PM, Pectoralis Major; SCOM, Sterno-Cleido-Occipito-Mastoideus; TLA, Triceps Lateral head; TLO, Triceps Long head; TP, Trigger Point; TU, Trapezius Upper, VAS, Visual Analogue Scale.

### Motor pattern and kinematics

**Figure 1:**
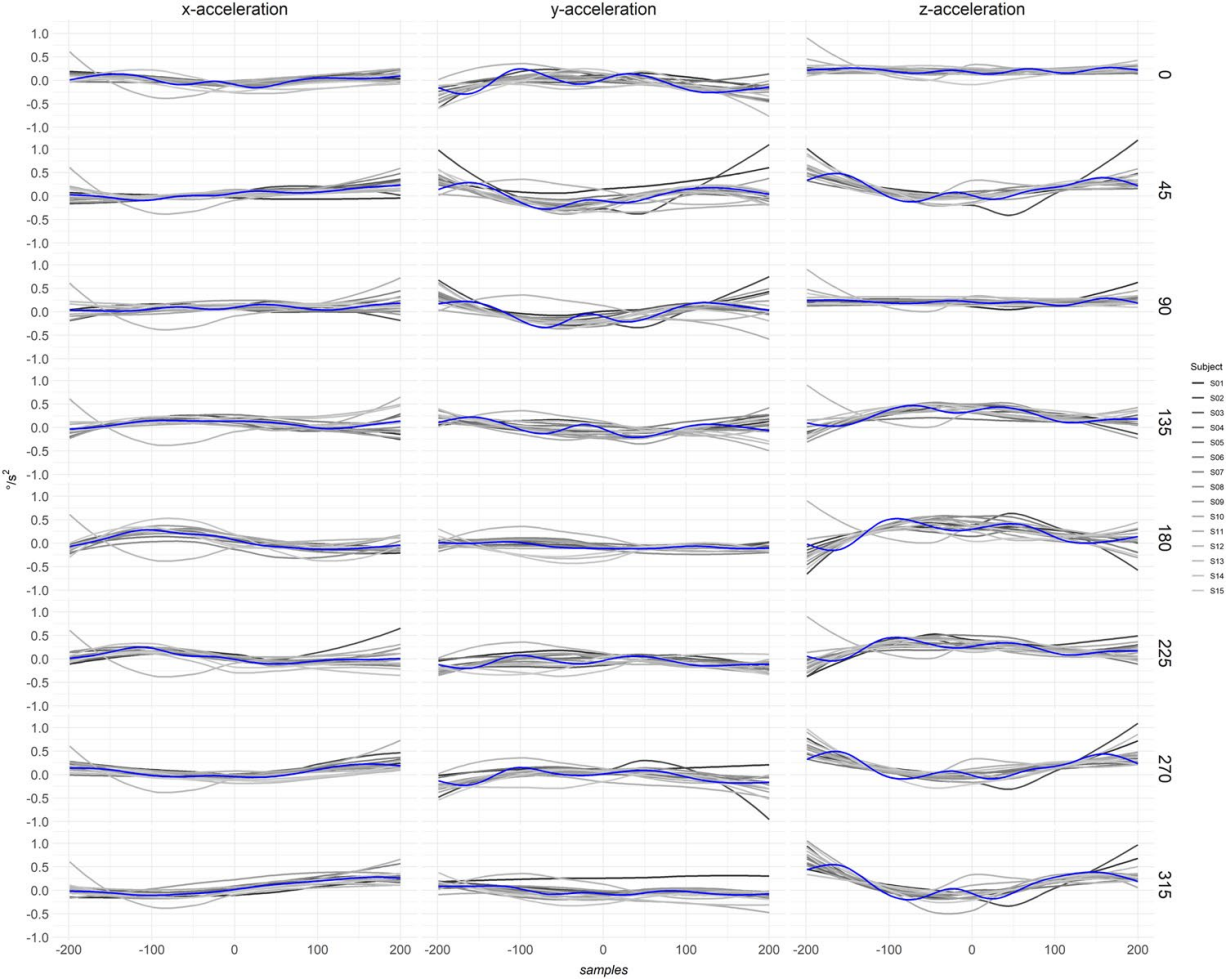
Time-profiles of *x-*, *y-*, *z-*accelerations of the wrist inertial sensor. In each column, the raw data of the kinematic variable is plotted versus the samples. The blue line represents the averaged value. The different number of samples from the one of the activation signals (see Fig. 3) is due to the different sampling frequency of the inertial sensor acquisition system. Each subject is represented by a line shaded in grey scale. Please note that the order of target is reported in degrees of a cartesian plane, therefore the order of target from top to bottom is 3, 2, 1, 8, 7, 6, 5, 4.

A representative example of the time profiles of *x-*, *y-*, *z-*accelerations for all subjects and angles is reported for the wrist inertial sensor as it was considered the most able to detect inter-individual kinematic variability (Fig. 1).

Figure 1 In terms of influence of TP on the COV of *x-*, *y-*, *z-*accelerations, roll, pitch, and yaw, there were no effects due to the number of TPs for all sensors and movement directions (Χ^2^ of all tests with P > 0.05). These results indicated that the experimental design allowed the subjects to perform the reaching tasks with similar kinematic and without different movement patterns despite the different distribution of TPs.

### Dimensionality and similarity across-subjects

The structure of motor modules varied among subjects, which shown that a different dimensionality was needed when applying the inflexion point method individually. For instance, two to five modules were detected (S01 = 3, S02 = 2, S03 = 3, S04 = 3, S05 = 4, S06 = 2, S07 = 3, S08 = 5, S09 = 3, S10 = 2, S11 = 4, S12 = 5, S13 = 4, S14 = 4, S15 = 3). The regression analysis shown the absence of any correlation between dimensions and number of TP (Pearson’s r = 0.10, P = 0.72) or the presence of active TP (Pearson’s r = 0.30, P = 0.26).

The curve of the VAF values averaged across subjects shown a change in slope at 3 factors (VAF = 0.77 ± 0.06, see Supplementary Fig. S1). In order to test Hypothesis 2, the 3 motor modules extracted afterwards for all the subjects were coupled according to their similarity. Despite the high inter-subjects variability, the average NDP (calculated using Subject-8 as reference) was 0.73 ± 0.14.

### Motor modules and directional tuning

**Figure 2:**
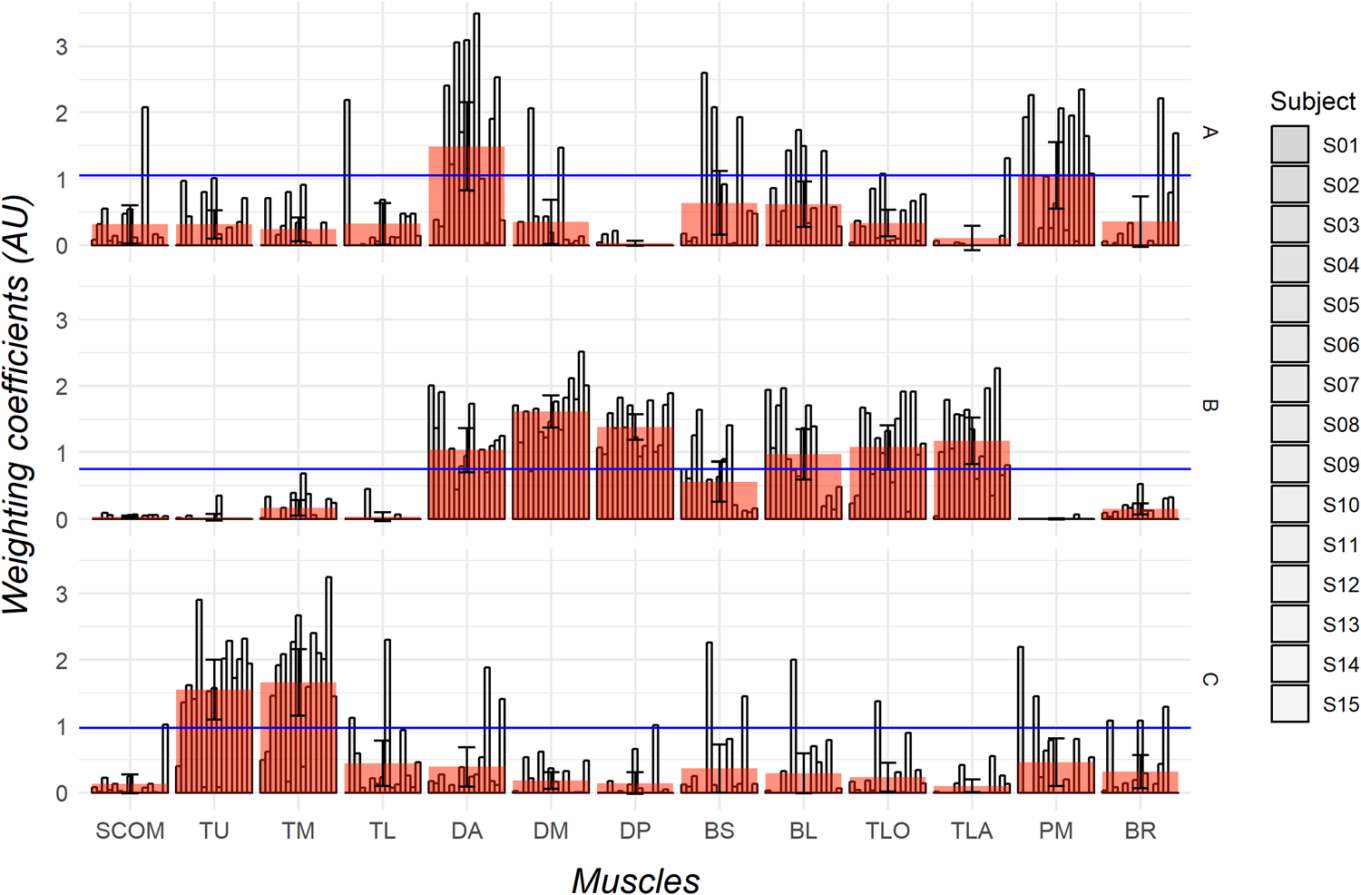
Motor modules retrieved from dimensionality analysis. Weighting coefficients are plotted for each subject and the mean with standard error in each synergy is superimposed for each muscle to show which are the dominant muscles for each synergy. Module A had the DA as dominant muscle, Module B had the DA, DM, DP, BL, TLO, and TLA muscle; and Module C had the TU and TM muscles. Note that despite the mean indicate an averaged dominant muscle, some subjects may have lower or higher weighting coefficients for that muscle (see Supplementary Fig. S2). Modules A, B, and C are described on the right. AU, Arbitrary Unit; BL, Biceps Long head; BS, Biceps Short head; BR, Brachioradialis; DA, Deltoid Anterior; DM, Deltoid Middle; DP, Deltoid Posterior; TL, Trapezius Lower; TM, Trapezius Middle; PM, Pectoralis Major; SCOM, Sterno-Cleido-Occipito-Mastoideus; TLA, Triceps Lateral head; TLO, Triceps Long head; TU, Trapezius Upper.

The 3 motor modules are reported in Fig. 2 across subjects and with a superimposed global value calculated as mean and standard error across muscles. Motor modules were arbitrarily called A, B, C and dominant muscles for each subject were established with respect to the criterion of a weighting coefficient 30% higher than the maximum value within each module (solid horizontal blue line in Fig. 2). The weighting coefficients were similar among male and females.

**Figure 3:**
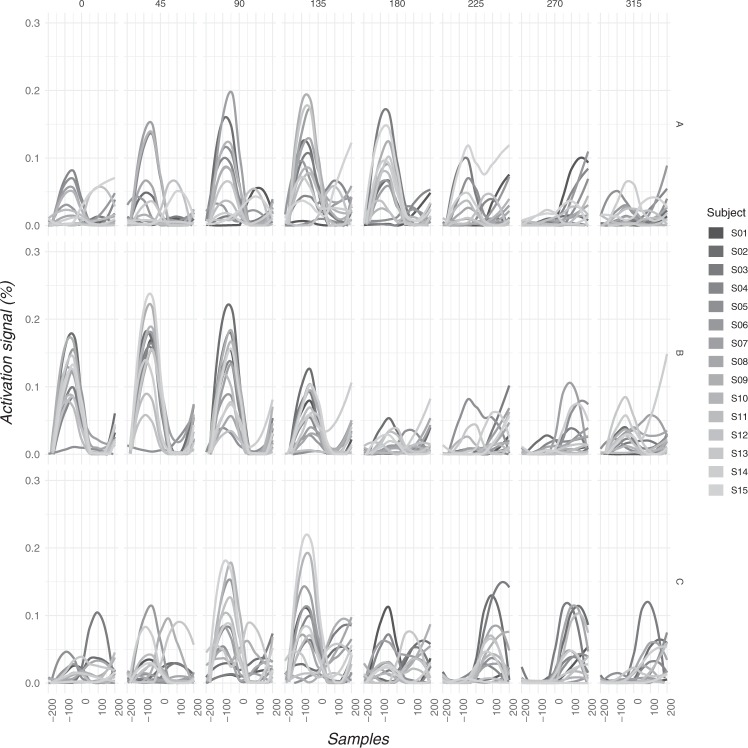
Time-profiles of the activation signals of all subjects for all motor modules and angles. Note that modules 2 and 3 overlaps for certain angles: this happened because some subjects achieved the criterion at 2 modules but 3 modules were extracted for all subjects in order to have comparable and meaningful data. Modules A, B, and C are described on the right. Please note that the order of target is reported in degrees of a cartesian plane, therefore the order of target from left to right is 3, 2, 1, 8, 7, 6, 5, 4.

Figures 2 and 3 displays the time profile of the activation signals of the 3 modules for each angle (directional tuning).

### Statistics

**Figure 4:**
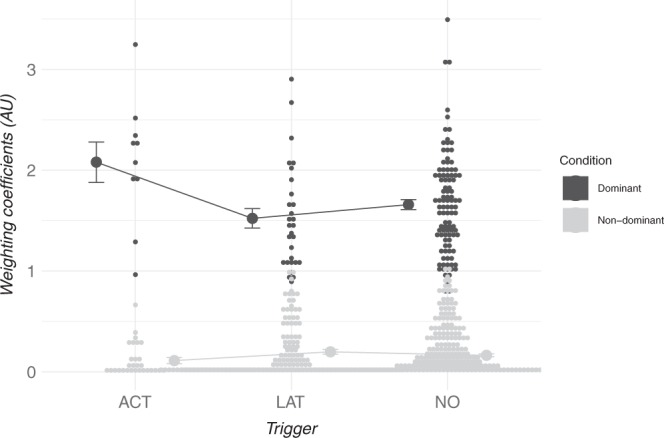
Second order interaction plot representing the influence of active trigger points on muscles in the dominant condition (black) while in the non-dominant condition (grey) there is no difference between muscles with or without trigger points. Small dots aligned above the three columns of trigger point presence conditions (ACT, LAT, NO) represent every single observation. Big black dots on the left and the big grey dots on the right of each column represented the mean values with error bars predicted by the mixed model. ACT, Active trigger point; AU, Arbitrary Unit; LAT, Latent trigger point; NO, Absence of trigger point.

The reduced model resulted in a significant interaction between TP presence and muscle condition on the weighting coefficients (AIC = 352.5, Χ^2^ (3) = 916.39, P < 0.001). The post-hoc analysis of the interactions revealed a significant difference when the muscles were in the dominant condition between ACT TP and LAT TP (Mean Difference (MD) = 0.6, 95%CI 0.32–0.78, P < 0.001) and between ACT TP and absence of TP (MD = 0.4, 95%CI 0.21–0.63, P < 0.001). The ES of this interaction was 0.46. No difference was observed in the dominant muscle between LAT TP and absence of TP (MD = − 0.1, 95%CI = −0.26–0.01, P = 0.05, ES = −0.12). When the muscle with TP was in the non-dominant condition, no differences were found between ACT TP and LAT TP (MD = −0.1, 95%CI = −0.20–0.06, P = 0.29), ACT TP and absence of TP (MD = 0, 95%CI = −0.17–0.08, P = 0.5) and LAT TP and absence of TP (MD = 0, 95%CI = −0.04–0.09, P = 0.44) (Fig. 4). The marginal and conditional R-squared of the reduced model were 0.79 and 0.80, respectively, thus indicating that both the fixed and the random effects explained a significant proportion of variance of the fitted model. The diagnostics run on this model indicated no significant deviation from the normality assumptions (see Supplementary Figs S5–S10).

**Figure 5:**
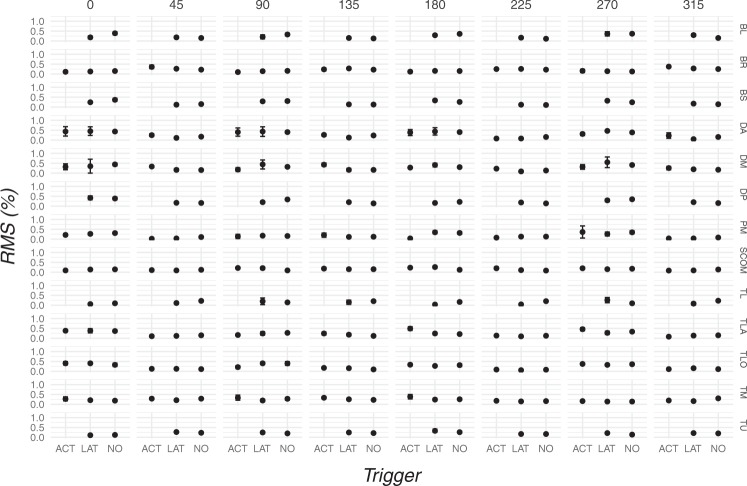
Mean and error bars of RMS of the three TP conditions (bottom) of each muscle (right) represented for every target (top). Increased muscle activities in presence of active TP were revealed for the TLA muscle at 180° and the DM muscle at 135°. A significant RMS decrease associated with active TP was detected for the PM muscle at 180°. Please note that the order of target is reported in degrees of a cartesian plane, therefore the order of target from left to right is 3, 2, 1, 8, 7, 6, 5, 4. ACT, Active trigger point; BL, Biceps Long head; BS, Biceps Short head; BR, Brachioradialis; DA, Deltoid Anterior; DM, Deltoid Middle; DP, Deltoid Posterior; LAT, Latent trigger point; TL, Trapezius Lower; TM, Trapezius Middle; PM, Pectoralis Major; RMS, Root Mean Square; SCOM, Sterno-Cleido-Occipito-Mastoideus; TLA, Triceps Lateral head; TLO, Triceps Long head; TU, Trapezius Upper.

The analysis of RMS of each muscle displayed no difference in muscle activity patterns as no interaction between presence of TP, angles and modules was detected (AIC = 2039.7, Χ^2^_(168)_ = 115.65, P = 0.99) (Fig. 5).

Two thousand grids simulating the presence of TP were randomly generated and only 7 (0.0035%) datasets gave a result within the 95% CI estimates derived from the original data. Therefore, the hypothesis that a significant result may arise from a spurious association between TPs detected with manual palpation and weighting coefficients of muscles within motor modules was rejected.

## Discussion

The present investigation on the influence of TPs on the modular control of reaching movements found a preliminary evidence that the weighting coefficients of dominant muscles hosting an active TP were higher than those of the dominant muscles hosting a latent TP or without TP, while no differences were detected among muscles in non-dominant condition. Furthermore, the regression analysis between dimensionality of modules structure and TP presence showed no correlations nor the analysis of RMS showed any influence of TP presence across muscles and angles. Previous literature already demonstrated an influence of TP at multi-muscular level on the muscle recruitment order during dynamic tasks^21,22^, even though it was still unclear whether a TP altered the EMG activity of the agonist muscle^16,17^ and whether these alterations influenced the modular organization of movement in terms of weighting coefficient of the involved muscle. This preliminary evidence expands the understanding of the topic of TPs for their etiopathogenesis and for their clinical and research management.

The analysis of the structure of motor modules revealed that the presence of TPs left the control of the modular architecture of shoulder girdle and upper limb muscles unaltered, as the 3 modules structure was similar to a previous study on anti-gravitational reaching task using the inflexion point method^25^. The number of extracted modules varied among subjects, suggesting that TP presence may alter the dimensionality of the individual modules structure. A recent review reported an inconsistent evidence of the influence of musculoskeletal pain on the number of modules extracted according to VAF values, with studies reporting a decreased, an increased or a similar number of modules^33^. The regression analysis reported herein showed no correlations between individual number of extracted modules and number of TP or presence of active TP. Despite heterogeneous dimensionality was present, it was probably unaffected by TP; however, further studies are needed to clarify this issue. Furthermore, none of the subjects showed an altered control of motor modules in contrast to what is seen with motor cortical damage^32^.

Despite the evidence of an alteration of weighting coefficients is inconsistent when muscle pain is induced experimentally^33^, the present results highlight for the first time that TPs influence how much a muscle is involved in a module. The model run on the influence of TP presence (ACT, LAT, NO) on the weighting coefficients, used to understand the amount of muscle involvement within a module confirmed our Hypothesis 2 that a TP altered the weighting coefficients of the muscle with the TP when it is involved in the motor modules as dominant. However, the absence of a difference in the non-dominant condition indicates that when a muscle participated in a motor module without a dominant role, the presence of TP left its weighting coefficient unaltered. This preliminary evidence shows that TP did not induce a pattern of co-contraction across muscles and, consequently, contradicts a previous study that attributed the increased activity of the antagonist muscle with TPs to a dysregulation of the reciprocal inhibition^20^.

Our results on RMS agree with previous studies that found no increased muscle activity in presence of latent TP^16,17^. Whilst other Authors reported an increased muscular activity of muscles hosting a latent TP^17,18,20^ their results are likely attributable to a higher selectivity of intramuscular EMG, compared to the bipolar superficial EMG in our study. As increased twitch force has been reported despite no changes in superficial EMG^35^, mechanomyography may be useful to study muscles with latent TP.

The higher activity of the agonist muscles with active TPs has been ascribed to a central alteration of muscle tone^36^ that leads to a higher recruitment of the associated alfa-motoneurons either at rest, as demonstrated by the presence of the SEA^15^, and during reaching tasks. Some authors have hypothesized an involvement of the Ia-inhibitory interneuron^20^, the Renshaw cells^19^ and the gamma-motoneurons^6^, while others have suggested a direct dysfunction occurring at the motoneuron soma^7^. Furthermore, other authors have pointed out that the motoneuron is also aided by an altered nociceptive pathway arising from lesions of the neural tissue^37^ or of the tissues segmentally related to the innervation level of the muscle with TP^8^ or from the direct compression of the nerve roots at the vertebral level, which the muscle with TPs segmentally belongs to^9^. Despite the fact that all the proposed spinal neurophysiological mechanisms point to an altered muscle tone causing an unpredictable muscle activation in presence of TPs, this preliminary study shows that the alteration is evident also at the level of motor modules, as it emerged only in the module when the muscle was dominant, becoming consequently an activity-driven alteration.

All the hypotheses on TP formation assume that a TP is born in its latent state after prolonged or unaccustomed exercise, low-load repetitive exercise, trauma or sustained stress that leads to muscle damage (or other nociceptive sources).

After a TP has formed, it acts as a peripheral nociceptive source and may start the abovementioned alterations of the spinal circuitry that, when fatigue arises, result in an overall increased EMG activity and decreased firing rate of the involved motor units^38^. This motor behaviour resembles what is observed during the motor adaptation to pain when the loss of force output due to a generalized reduction of discharge rate^39^ in the painful muscle is compensated via a heterogeneous recruitment of additional motor units^40^ in the acute phase, and a reduced complexity of motor unit recruitment^41^ in the chronic phase. These mechanisms explain the increased activity of agonist muscles with active TPs, as the criterion discriminating active from latent TPs is the reproduction of familiar pain. In contrast, the increased EMG activity occurring in the muscle with latent TP has been linked to the recruitment of additional motor units that allows the achievement of a similar force output^17^. This concept is supported by several studies documenting a similar force in subjects with and without latent TPs during isometric or dynamic contractions tasks^16,17,21^. The present study enriches the perspective on TPs as they increased also the weighting coefficient of the dominant muscle of a module. Therefore, the contraction of muscle fibres typical of TPs may couple with the redistribution of muscles weighting coefficient within a module that recruits more the muscle with active TP. In this new perspective, TPs may represent the epiphenomenon of a motor re-organization occurring at the level of motor modules. Despite the understanding of the mechanism originating the dysfunctional motor module is beyond the observational purpose of this experiment, we can speculate that a common dysfunctional motor module may present across patients with different clinical scenarios and the associated TPs. For instance, the presence of specific TPs in individuals will emerge as the result of a complex interaction among the dysfunctional motor module, the individual characteristics (physical and cognitive) and the environmental context where the movement is executed. Accordingly, the heterogeneity of the alteration of motor modules in presence of muscular pain^33^ may occur because researchers have focused their attention on finding similar motor alteration in patients with similar pain syndromes. However, it may be the case that a common dysfunction of motor modules may have heterogeneous clinical manifestations. This hypothesis may be tested by demonstrating that a sample of subjects with various nonspecific musculoskeletal disorders of the upper quadrant, such as neck, shoulder and arm pain, share common dysfunctional motor modules during the reaching task performed in this experiment. Therefore, future research in the field should consider the exploitation of dysfunctional motor modules as a new field of inquiry in presence of TP and musculoskeletal pain.

In clinical practice, the clinician usually selects the muscle supposed to be involved in the matching between the patient’s pain quality and history, such as aggravating movements or postures, with the body map of referred pain pattern^42^. Once the muscle has been selected, then the clinician follows the muscle palpation to detect the typical clinical signs of the TP (e.g. local tenderness, taut band, patient’s pain recognition and referral, local twitch response)^3^. If the muscle palpation gives positive findings, then the muscle is diagnosed as having an active TP. The treatment is often based on an iterative treat-and-reassess process wherein the clinician re-directs the treatment to other muscles, using the abovementioned reasoning, until the patient’s symptoms are modified/disappeared. The treatment is often supported with stretching and strengthening of the muscle involved^2^. This study provides preliminary evidence that the analysis of motor modules may reveal which is the dysfunctional muscle and whether the treatment has been effective in the restoration of an unaltered motor control. Furthermore, the understanding of the involved motor module may inform the selection of exercises that recruit specifically the affected motor module.

The potential limits of the study are related to the detection of TP with only one examiner and to the small sample size. The bias in palpating and detecting TPs was handled refitting the same model using a random generation of the observed TP frequencies. Even though it was not possible to analyze all the possible combinations, only a small and statistically significant proportion of the 2,000 generated datasets produced similar results as ours, supporting that the presence of any spurious association was unlikely. A small sample size may produce an underpowered study for the reported effects. However, the mixed model approach had satisfactory marginal and conditional R-squared (e.g. the degree to which the variables set as fixed or random effects explain the variance of the model). Furthermore, the ES for the difference between active TP versus latent or no TP was moderate. Another limitation of the study was related to the motor module decomposition that was obtained merging only two repetitions for each movement even though a higher number of repetitions is suggested to improve decomposition quality^43^. Therefore, further studies with bigger samples, established using the R-squared and ES reported in this article, and more repetitions are needed to test the Hypothesis 2 of this study that, at this time, suffered from an over-inflation of the results. Finally, only superficial muscles were studied as their palpatory examination of TP is comparable to the diagnosis made by ultrasound^44^. It is possible that using intramuscular EMG and ultrasound identification of TP also in deep muscles may have revealed further findings, as superficial EMG may be biased by cross-talk effects of the muscle underneath the ones studied. However, at this time we preferred to not overcomplicate an initially explorative study.

In conclusion, the present study reported an increased weighting coefficient of muscle hosting active TPs when the muscle was dominant according to the modular control of movement. Further, TPs did not alter the dimensionality of modules structure nor muscle activity. The results expand the perspective on TP at a multi-muscular level suggesting the absence of a co-contraction pattern. The analysis of motor modules may assist clinicians in measuring the effectiveness of therapeutic interventions directed to TP.

## Methods

### Study design and setting

A cross-sectional study was performed at the research laboratory of the Campus of Savona, Department of Neurosciences, Rehabilitation, Ophthalmology, Genetic, Maternal and Child Health (DINOGMI) of the University of Genova. The study received approval by the Regional Ethical Committee (Liguria - P.R. 095REG2015). All participants signed an informed consent and were allowed to withdraw from the study at any time. The experiment was performed in accordance with relevant guidelines and regulations.

### Study population

Fifteen volunteers aged 18–50 years, were recruited to form a convenience sample. As the focus of the experiment was to detect the influence that TPs have on motor control, people with or without musculoskeletal disorders were considered. Subjects were excluded if they showed relevant comorbidities (tumors, central or peripheral neurological disease, rheumatic diseases, previous surgery to the spine or upper limb, cardiovascular diseases) or were currently taking muscle relaxants, or other medications possibly influencing muscular activity or pain.

### Baseline variables

Subjects filled up a self-report questionnaire to collect the following demographic variables: gender, age, weight, height, pain intensity and location on a body map.

To measure health-related quality of life in each subject, the following cross-culturally adapted and validated Italian questionnaires were administered: the EuroQoL Index (EQI) for the general health status^45^, the Visual Analogue Scale for pain intensity^46^, the Neck Bournemouth Questionnaire for neck pain functioning and anxiety^47^, the QuickDASH for function of neck and upper limb^48^, the Tampa Scale for Kinesiophobia to assess avoidance beliefs related to movement^49^ and the Pain Catastrophizing Scale to assess biased beliefs regarding pain experience^50^.

### Trigger point detection

An experienced examiner (TG) detected the presence of active and latent TPs using the criteria proposed by the most recent consensus on TP diagnosis^3^. The TP palpation procedure was based on the Anatomical Landmark Framework (ALF) lines used to avoid electrode positioning over the innervation zone (*see Electrode positioning and EMG recordings paragraph*). During the palpation of each muscle, the following criteria were tested for:

1. spot tenderness present in a taut band,
2. familiar (active TP) or unfamiliar pain (latent TP) elicited by palpation of the tender spot
3. referred pain elicited by palpation of the tender spot,
4. local twitch response (visible or felt under the fingertip) elicited during snapping palpation.

A muscle was considered to host an active TP when all the criteria were met. The TP was labelled as latent when the first and fourth criteria were respected, and the second criterion was scored for unfamiliar pain^3^. For a latent TP, the presence of the third criterion was considered not relevant.

Despite the fact that aforementioned criteria provides an acceptable inter- and intra-rater reliability in the identification of TPs in the upper quadrant^51^, a statistic was performed to avoid any biased identification of TPs due to the presence of only one examiner (*see Statistical analyses paragraph*).

### Experimental task

**Figure 6:**
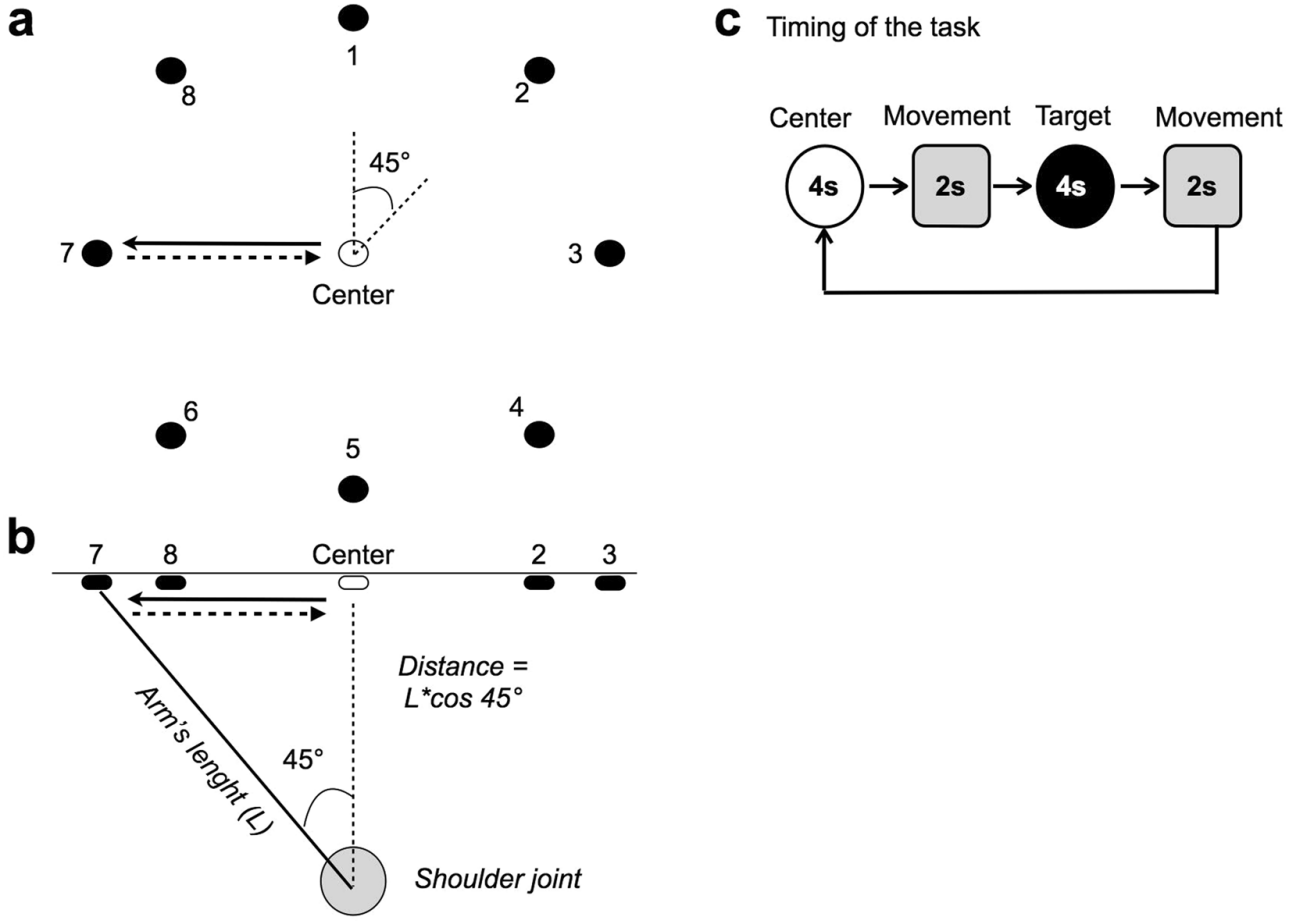
Experimental setup. (**a**) The wood panel had customized rails to adjust the radius of the circumference according to the distance between the shoulder joint and the central target to account for the inter-individual variability of arm’s length. (**b**) Geometric description of the criterion used to set the distance between the shoulder joint and the central target. The subjects had to reach each target with a straight elbow whilst the hand moved approximately 45° away from the initial position. (**c**) The task rhythm was paced in four timeframes. In the first, the subject pushed the central button for 4 seconds while keeping the arm on the horizontal plane. In the second, the subject reached one radial target in about 2 seconds. In the third, the subject pressed the radial button for 4 seconds. In the fourth, the subject moved back to press the central target for about 2 seconds and then relaxed the arm.

Subjects performed an upper limb reaching task in two ways: circular and random. A wood panel supported 9 targets that the subjects were requested to reach in a timely manner (Fig. 6a–c). Eight targets were arranged in a circumference at 45° intervals, whilst the ninth target constituted the centre of the circumference (Fig. 6a, see also Gizzi *et al*.^52^). The height of the subject’s seat was regulated to align the shoulder joint with the central target. The criterion established to adjust the radius was that subjects had to reach each target with a straight elbow whilst the hand moved approximately 45° away from the initial position. (Fig. 6b). For each target, the acquisition started with the subject sitting straight on a chair with their knees 90° flexed. An initial training session was administered to let the subject familiarize with the experimental setting and the task rhythm that was organized in four periods (Fig. 6c). For the circular task, the subjects reached all the targets in a clockwise direction consecutively starting from target 1. For the random task, the order of presentation of targets was randomized for all participants. In both tasks, subjects performed one repetition for each target. A rest period was provided after each set of movements to prevent fatigue. In the meantime, data was saved on a computer hard-drive for offline analysis. Only the EMG produced during transitions (i.e. second and fourth periods, Fig. 6c) was used for motor module decomposition.

### Electrode positioning and EMG recordings

Pairs of Ag/AgCl electrodes (15 × 15 mm bipolar electrodes, Spes Medica, Genova, Italy) were positioned on the following 13 muscles of the right side (all the subjects were right-handed): upper, middle and lower trapezius (TU, TM and TL, respectively), anterior, middle and posterior deltoid (DA, DM, and DP), pectoralis major (PM), medial and lateral head of the biceps brachii (BS, BL), lateral and long head of the triceps brachii (TLA,TLO), brachioradialis (BR), and the sternal head of the sterno-cleido-occipito-mastoideus (SCOM). Skin was prepared by gentle abrasion with abrasive paste (Every, SpesMedica, Genova, Italy) and cleaned with water. Electrodes were positioned along the ALF following established criteria^53^ to avoid positioning over the innervation zone. The ALF was marked with a blue pencil for all the muscles on each participant’s skin at the beginning of the experiment and served also for TP detection. The signal quality achieved using these criteria varies from good to excellent for all muscles^54^. The surface EMG acquisition (EMG-USB, OTBioelettronica, Turin, Italy) was synchronized with kinematic recordings. The EMG segmentation and computation of motor modules were performed in MATLAB (Matlab 2016b, the Mathworks, Natick, Massachusetts) via a custom made script.

### Kinematic data recording

Subjects were equipped with inertial sensors (Xsens Technologies B.V, Enschede, The Netherlands) to record the motion of the arm using x-, *y-*, and *z-*acceleration profiles and 3D articular angles (roll, pitch, yaw). Inertial sensors were positioned on the posterior aspect of the distal third of the forearm and of the arm respectively, the top of the acromion, and the Lewis sternal angle.

The 3D characterization of movement allowed to select the portion of movement from which extracting the EMG signal (Fig. 6c). The Coefficient of Variation (COV) was estimated for each kinematic variable and used to assess whether the presence of TP altered the kinematic of the movement across subjects (*see Statistical analysis section*).

### Preprocessing, segmentation and normalization of EMG signal

EMG data was band-pass filtered (10–450 Hz, 2^nd^ order Butterworth filter) and then high-pass filtered (50 Hz, 2^nd^ order Butterworth filter) to attenuate movement artifacts^55^.

EMG segmentation was based on the start and the end of the movement, detected when the speed value exceeded the reference value of the standard deviation of the rest phase by over 5%. The influence of gravity on the EMG was corrected according to d’Avella *et al*.^55^. Briefly: the average rectified EMG value for each muscle was computed in two 300 ms windows (one prior to, and one after the completion of the movement). The two baseline values were linearly interpolated across the length of the movement, and the line was subtracted from the signal envelope (see below) on a sample-by-sample basis. The EMG data of each movement was first concatenated, and then full-wave rectified and low-pass filtered (5 Hz, 4^th^ order Butterworth filter); the tonic activity for each movement was subtracted and individual movements were resampled to 200 samples^52^. Since NMF only works with non-negative data, the EMG values falling below zero after tonic activity subtraction were padded to zero^55^. For each movement, the EMG was normalized using the average of the global maximum of the two trials after gravity compensation. The data from two trials for the same target were averaged. The amplitude of the muscular activity was estimated with the Root Mean Square (RMS) of the normalized EMG signal for each muscle. The RMS indicated the degree of muscular activation and is termed “muscle activity” throughout the manuscript.

### Muscle synergies analysis and non-negative matrix factorization

The extraction of muscle synergies with Non-negative Matrix Factorization (NMF) followed well-established procedures^28,52^. The NMF algorithm^34^ was used to extract the motor modules from the segmented EMG signal. Each motor module is composed by a set of weightings (*S*, indicating the extent of mutual activation of different muscles within one synergy and termed “weighting coefficients” throughout the manuscript) and one of activation signals (*P*, which quantify the extent of recruitment of a given set of weightings over time) according to (1):

1 $$X(k)\approx {X}_{r}(k)=S\cdot P(k)$$

Since, while using NMF, the dimensionality of the solutions space is not known a priori, a number of synergy variable between 1 and 12 was computed and the dimensionality was chosen according to the following criterion: for each NMF run, the reconstruction quality of the original EMG by means of the extracted muscle synergies was assessed as the Variance Accounted For (VAF), defined as:

$$[{\rm{VAF}}=1-{\rm{SSE}}/{\rm{SST}}]$$

where SSE it the Sum of Squared Error (computed as the difference between the reconstructed EMG signal and the original EMG data) and indicates the unexplained variance. SST is the Total Sum of Squares (calculated as the difference between each observation of the EMG data and its mean) and indicates the variance explained. The number of synergies to extract was determined using the inflexion point (e.g. change in slope) of the VAF curve averaged across subjects^25^. The dimensionality was also determined for each subject separately and correlated using a regression analysis with the number of detected TP and with the presence or absence of active TPs.

The NMF was initialized with a seeding method of random generation of non-negative matrices and was run 10 times. The run with the best reconstruction quality (*see below*) was selected for further analyses.

The similarity of muscle weightings across subjects was assessed with the average Normalized Dot Product (NDP), calculated as the scalar product of two vectors of motor modules divided by the product of their norms^30,52^. Each subject was iteratively isolated and used as a reference to compute the average NDP for all the others. The highest value was retained.

### Statistical analyses

The free statistical software R version 3.4.1^56^ was used for the statistical analyses. Demographic variables and questionnaire scores were reported as mean, standard deviation (SD), minimum and maximum values. The presence of active or latent TPs or their absence was reported as absolute frequency indicating the muscle involved for each subject.

The motor modules were summarized according to the data in matrix S (weights of motor modules) and matrix P (activation signals) to depict the structure of the synergies and their preferential activation in space. For the matrix S, a muscle was considered dominant for each subject in each module when its weight exceeded a threshold value calculated in percentage of the maximum weighting coefficient within each module^57^, which in this study was set at 0.3. This kind of muscles labelling was needed to couple the presence of TP with the role that a muscle has within a module, as a TP may influence the weighting coefficients of a muscles either dominant or not in a module. For matrix P, the time-profile of module activation was displayed individually for each angle and module. The number of modules for each subject was correlated with the number of total TP and the presence of active TP using a linear regression analysis (Hypothesis 1).

The potential bias of the repeated measurements due to the study design and data extraction method was handled using a linear mixed model statistic performed with the package lme4 version 1.1-13^58^ which allowed to take into account the variance due to the presence of multiple within subject factors (subject ID, muscles, motor modules, targets, sensor position). The model fit was tested with the Likelihood Ratio (LR) test of significance using the Maximum Likelihood (ML) estimator. The model with a significant Chi-squared (Χ^2^) test and the lowest Akaike’s Information Criterion (AIC) was retained for the analysis of the fixed effects. Significant higher order interactions were analysed with the least square means method with Kenward-Roger approximation of degrees of freedom using the package lmerTest version 2.0-33^59^. The marginal and conditional R-squared were used as a measure of the proportion of variance of the model explained by, respectively, the fixed and random effects. Calculation was performed using Kagakawa and Schielzeth’s method for mixed models implemented in the MuMIn package version 1.40.0^60^. The Effect Size (ES) was calculated using the simr package version 1.0.5^61^. The P value was set to 5%. For the model diagnostics the normality assumptions were tested inspecting quantile-quantile plots of the residuals and of each level of the random effects structure^62^. The model linearity was deemed acceptable when the difference between the fitted values and the residual values was close to zero. The independence among fixed effects was tested using the Variance Inflation Factor (VIF); a VIF lower than 2 was needed to avoid collinearity, and if the VIF was higher than 2 the related fixed effect was dropped from the model and the analysis of collinearity was re-run. Homoskedasticity assumption was confirmed when Levene’s test^62^ on the model residuals was not significant.

An alteration of the subject’s kinematic that may bias the conclusions drawn about the EMG decomposition was analysed after dichotomization of the subjects into two groups, classified as having active TP or not, respectively. A mixed model approach was used on the dependent kinematic variables (COV of *x-*, *y-* and *z-*accelerations, and roll, pitch and yaw) derived from the inertial sensors of each subject. For each kinematic variable, the group presence of TP (2 levels: ACT, NO) was treated as fixed effects while ID (15 levels), targets (8 levels: 0, 45, 90, 135, 180, 215, 270, 315), and sensor position (4 levels: sternum, shoulder, arm, and forearm) were treated as random effects. A non-significant Χ^2^ test indicated the lack of kinematic alterations.

For the statistic of matrix S, Hypothesis 2 was tested using a mixed model approach on the weighting coefficients, with presence of TP (3 levels: ACT, LAT, NO), muscles condition (2 levels: DOMINANT, NON-DOMINANT) and modules (3 levels: A, B, C) as fixed effects and ID (15 levels) and muscles (13 levels) as random effects. Subject ID was not considered a random effect as its variance was 0. There was a significant interaction between TP, muscles condition and motor modules (AIC = 301.26, Χ^2^_(9)_ = 980.58, P < 0.001, see Supplementary Fig. S3). However, the diagnostics revealed VIF values higher than 2 for either the single effects and for their interactions, therefore the model was re-run without the predictor module. Hypothesis 3 was tested with a mixed model approach on RMS values, with presence of TP (3 levels: ACT, LAT, NO), muscles (13 levels) and targets (8 levels) as fixed effects and ID (15 levels) as random effects.

In order to overcome the possible bias of the identification of TPs arising from the manual palpation executed by only one assessor, the analysis on matrix S was repeated with a random generation of 2,000 datasets of the TP presence to test Hypothesis 4. An acceptable result was considered when less than 5% of the random generated datasets gave results similar to those observed in the original data.

## Supplementary information

Supplementary materials

## Data Availability

The authors declare that data and materials are available to readers upon reasonable request.
