# Supplementary material for: Myofascial trigger points alter the modular control during the execution of a reaching task: a pilot study
Source: Sci Rep. 2019 Nov 5;9:16065. doi: 10.1038/s41598-019-52561-3 (PMC6831581; doi:10.1038/s41598-019-52561-3)
Supplement: Supplementary file 1 — Supplementary materials [file 41598_2019_52561_MOESM1_ESM.pdf]

Title:

Myofascial trigger points alter the modular control during the execution of a reaching task: a pilot study.

Authors:

Geri Tommaso *§, Department of Neuroscience, Rehabilitation, Ophthalmology, Genetics, Maternal and Child Health (DINOGMI), University of Genova, Campus of Savona, Italy. Via A. Magliotto, 2 – 17100 Savona (SV). mobile +39 340 0623614. phone/fax: +39 019 860250. e-mail: tommaso.geri@gmail.com

Gizzi Leonardo §, Institute for Modelling and Simulation of Biomechanical Systems, Continuum Biomechanics and Mechanobiology Research Group. University of Stuttgart, Germany. Phone/fax: +49 (0)711/68560044. e-mail: Leonardo.gizzi@mechbau-uni-stuttgart.de

Di Marco Anna, Department of Neuroscience, Rehabilitation, Ophthalmology, Genetics, Maternal and Child Health (DINOGMI), University of Genova, Campus of Savona, Italy. phone/fax: +39 019 860250. e-mail: annadm.ondequadre@gmail.com

Testa Marco, Department of Neuroscience, Rehabilitation, Ophthalmology, Genetics, Maternal and Child Health (DINOGMI), University of Genova, Campus of Savona, Italy. phone/fax: +39 019 860250. e-mail: marco.testa@unige.it

* : Corresponding Author

§ : These Authors contributed equally to the work

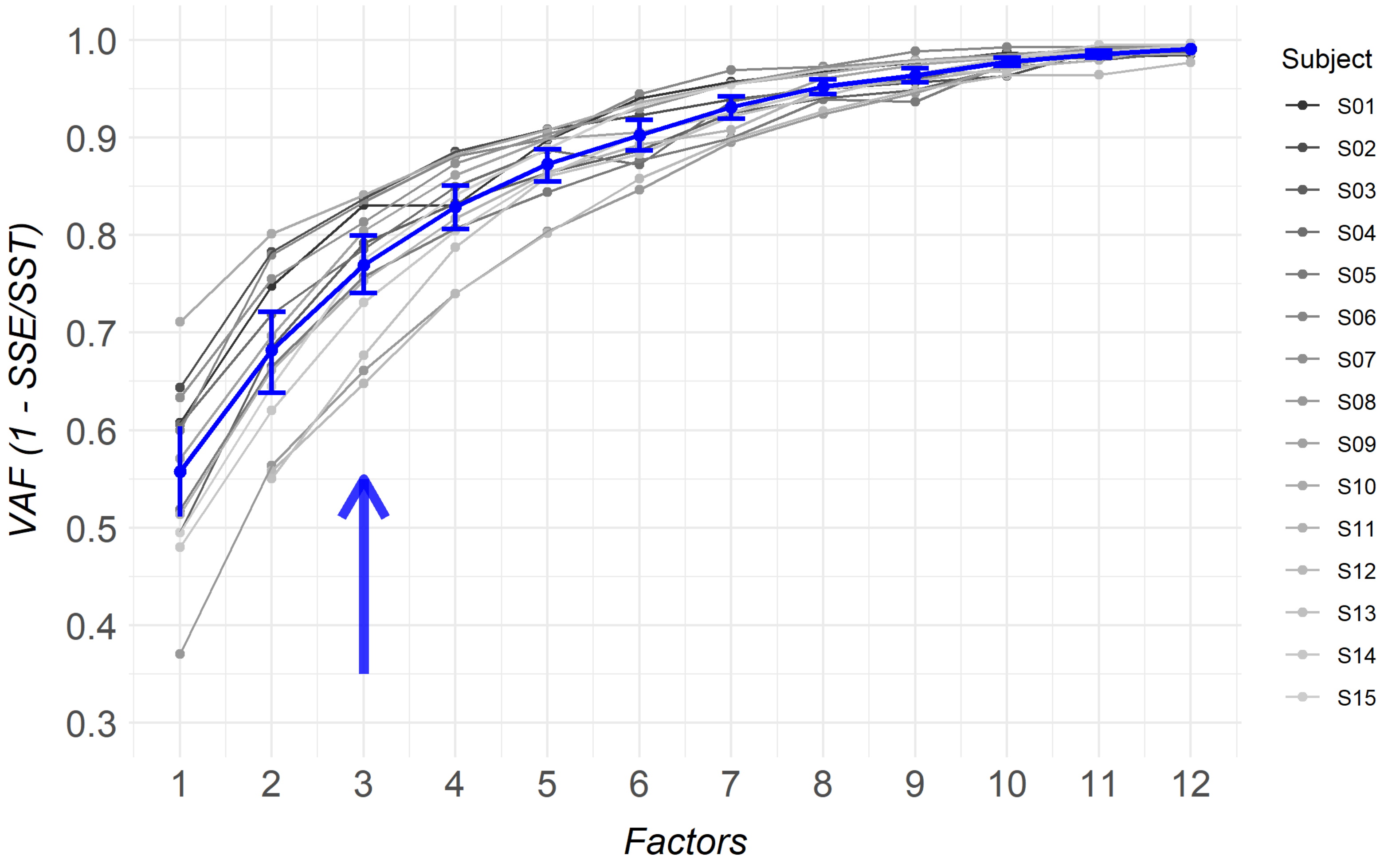

Supplementary Figure S1. VAF (Variance Accounted For) index - All subjects. The blue arrow indicates the inflexion point of the curve of the averaged VAF values (blue).SSE, Sum of Squared Error; SST, Total Sum of Squares.

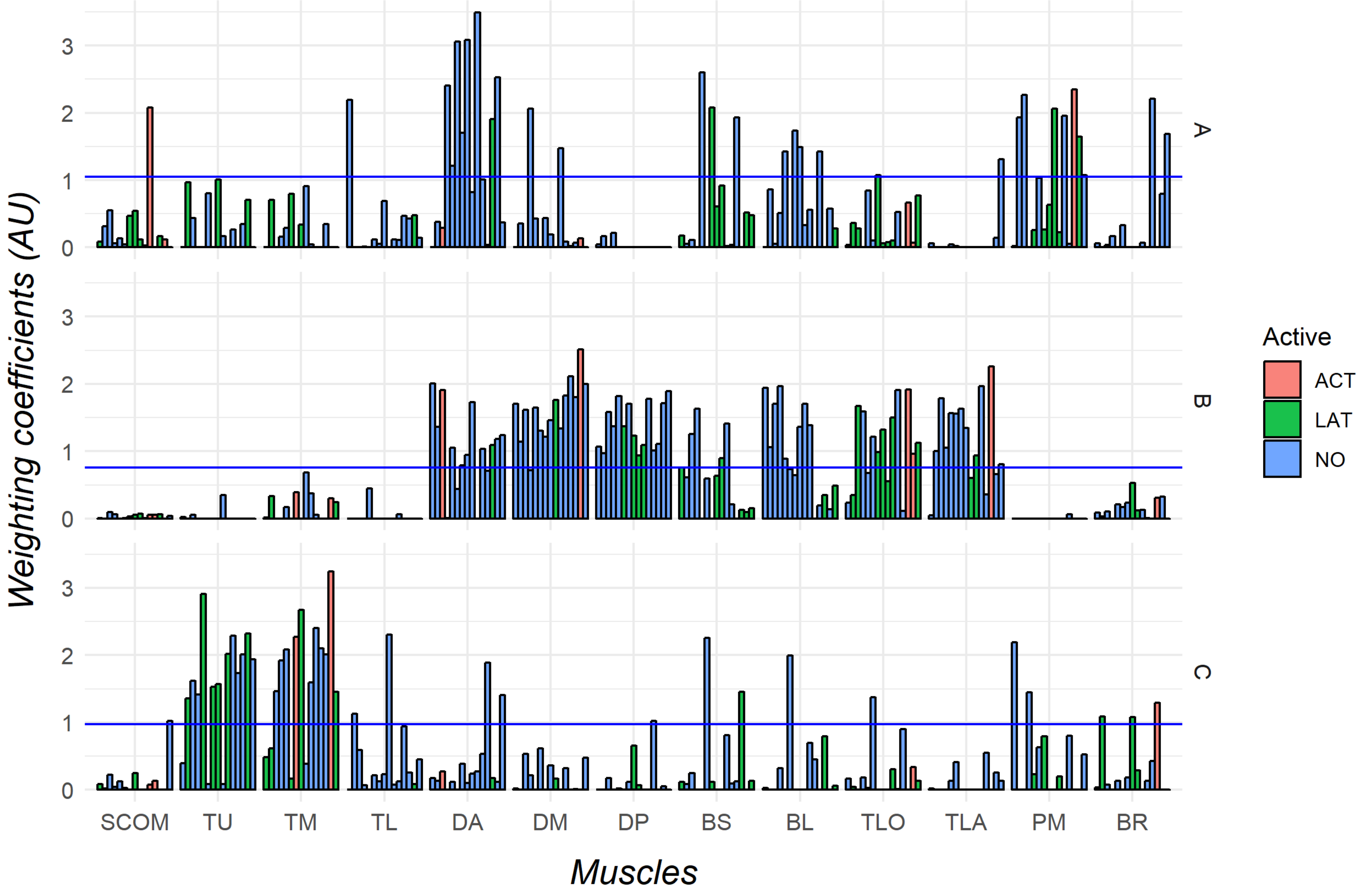

Supplementary Figure S2. Motor modules (A, B, C) coded according to the presence of active, latent or no trigger point. ACT, Active trigger point; AU, Arbitrary Unit; BL, Biceps Long head; BS, Biceps Short head; BR, Brachioradialis; DA, Deltoid Anterior; DM, Deltoid Middle; DP, Deltoid Posterior; LAT, Latent trigger point; TL, Trapezius Lower; TM, Trapezius Middle; PM, Pectoralis Major; SCOM, Sterno-Cleido-Occipito-Mastoideus; TLA, Triceps Lateral head; TLO, Triceps Long head; TP, Trigger Point; TU, Trapezius Upper.

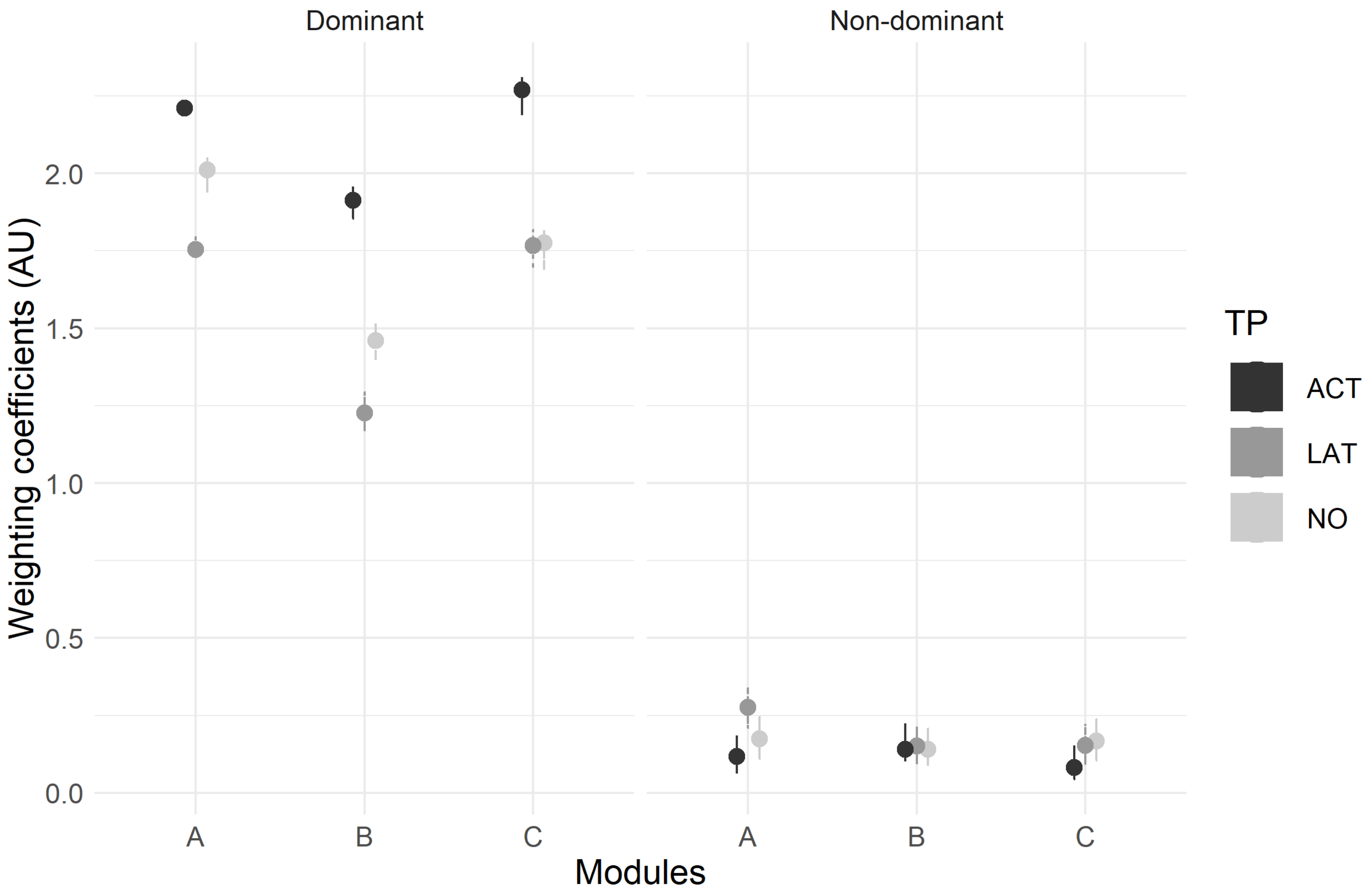

Supplementary Figure S3. 3th interaction among trigger point, muscle condition and modules on weights. Graphical representation of the full model. The model was discarded as VIF (Variance Inflation Factor) values were higher than the selected reference value (2) for the factor module. A, Module A; B, Module B; C, Module C; AU, Arbitrary Unit; ACT, Active trigger point; LAT, Latent trigger point; NO, Absence of trigger point.

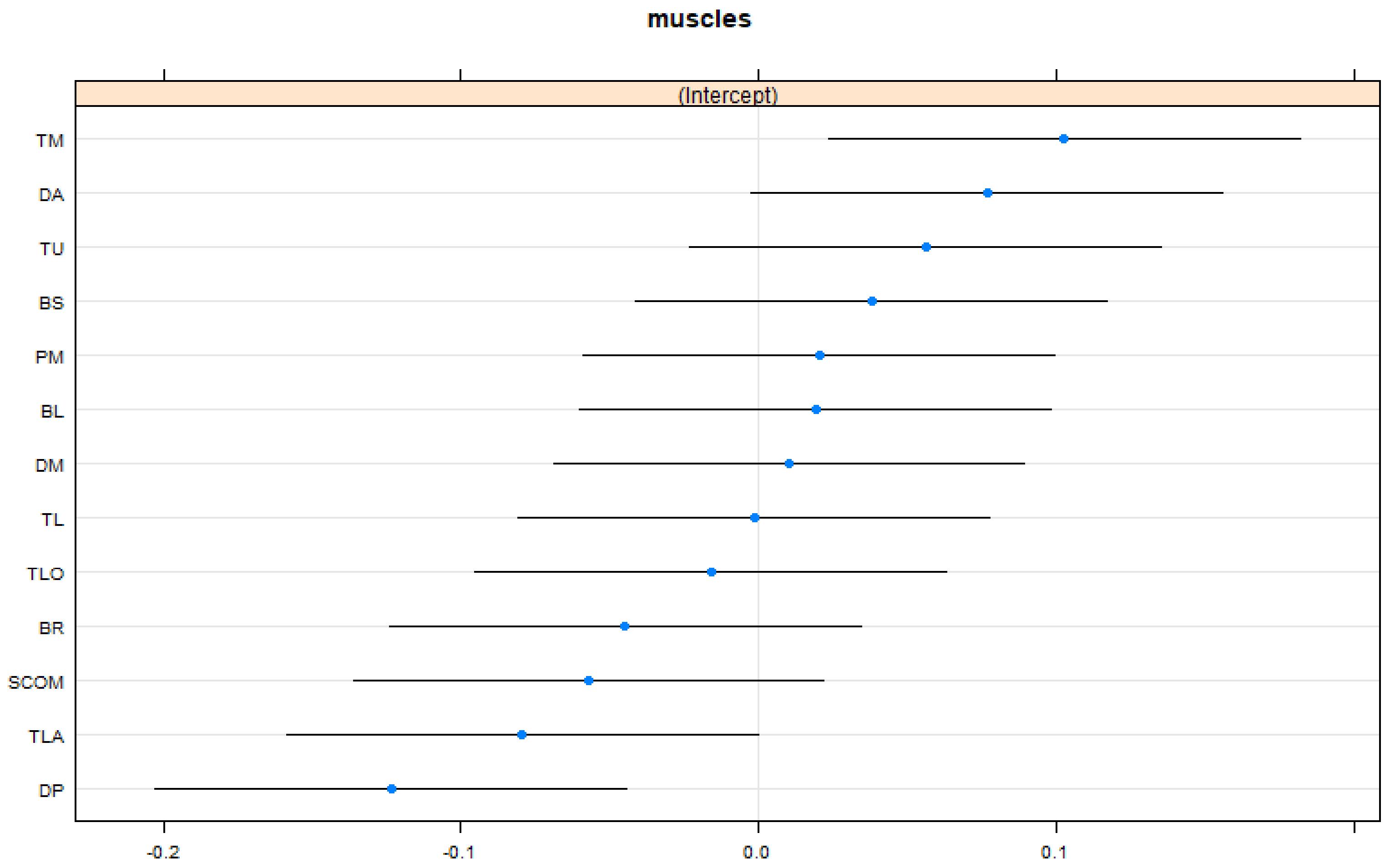

Supplementary Figure S4. Random effects of the muscles in the reduced model for interaction between TP presence and muscle condition. BL, Biceps Long head; BS, Biceps Short head; BR, Brachioradialis; DA, Deltoid Anterior; DM, Deltoid Middle; DP, Deltoid Posterior; LAT, Latent trigger point; TL, Trapezius Lower; TM, Trapezius Middle; PM, Pectoralis Major; SCOM, Sterno-Cleido-Occipito-Mastoideus; TLA, Triceps Lateral head; TLO, Triceps Long head; TP, Trigger Point; TU, Trapezius Upper.

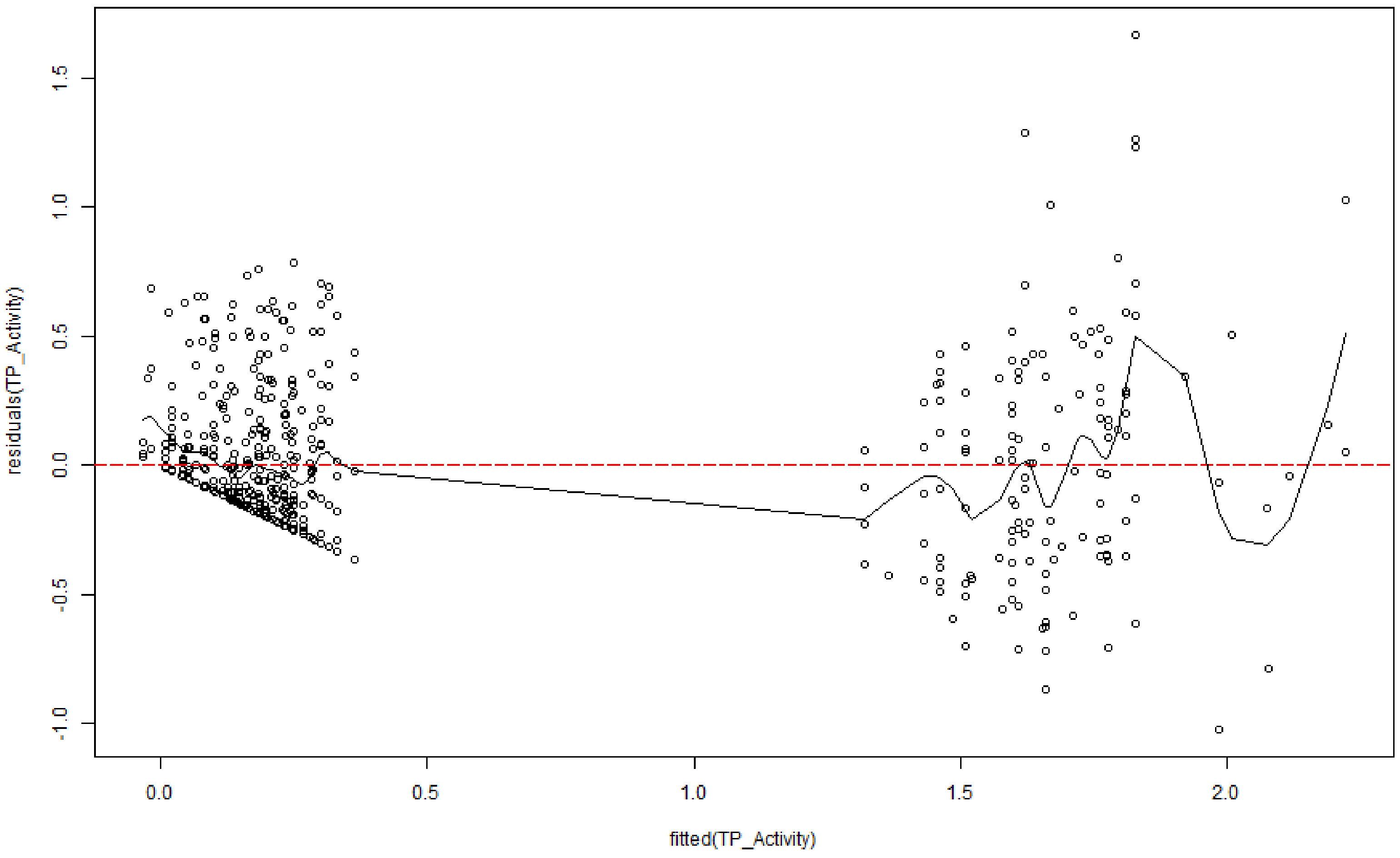

Supplementary Figure S5. Linearity of reduced model

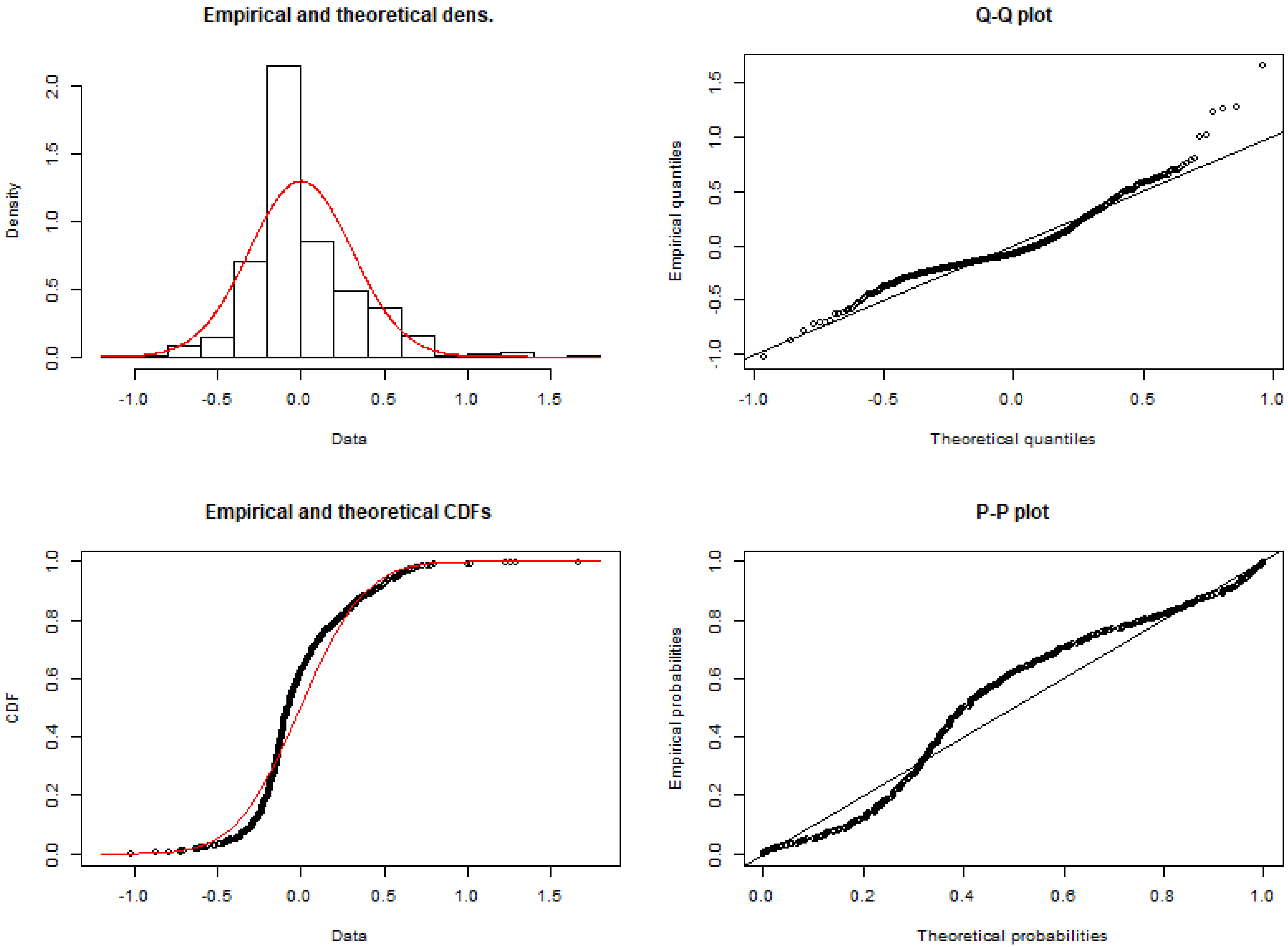

Supplementary Figure S6. Normality of reduced model

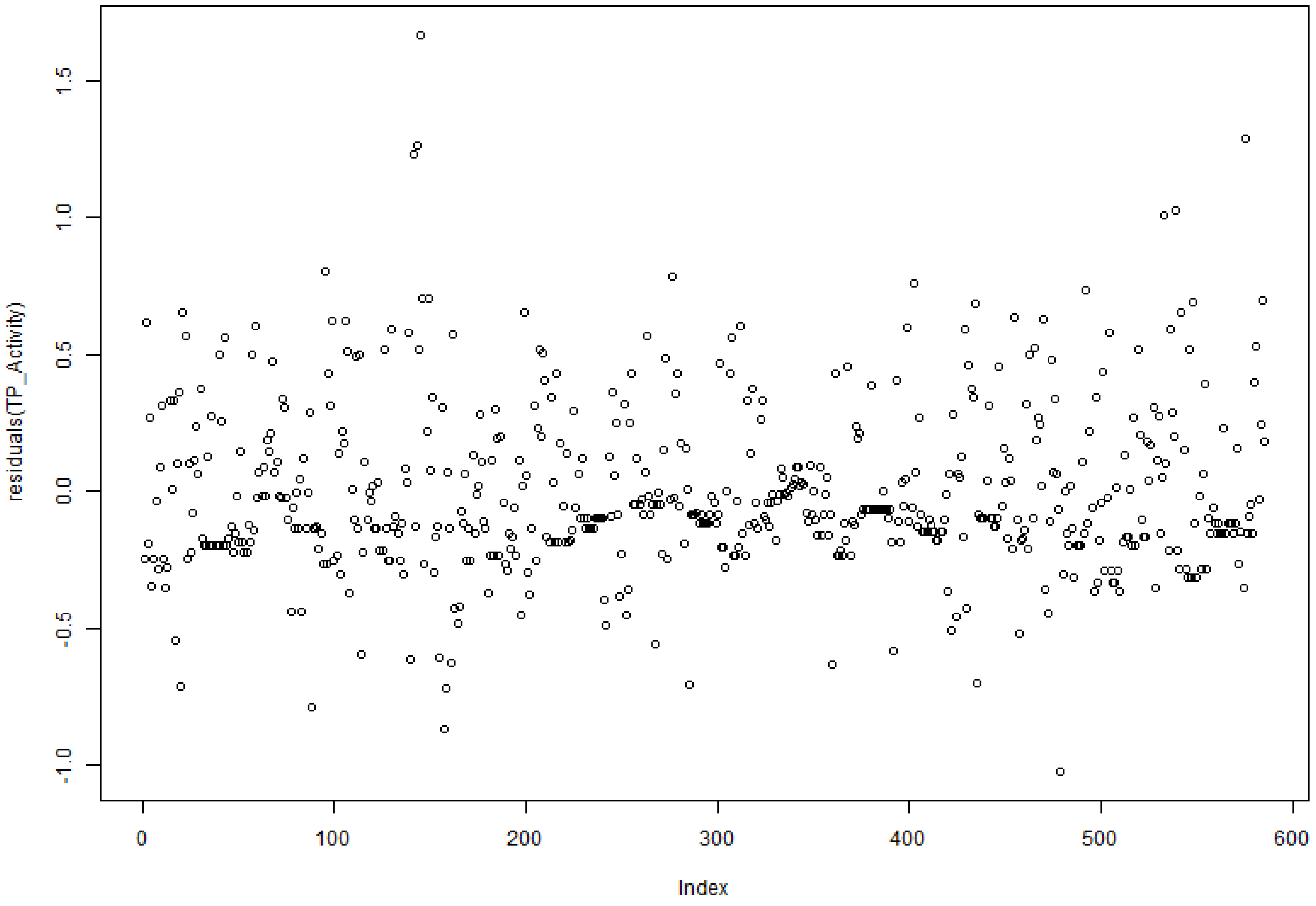

Supplementary Figure S7. Homoskedasticity

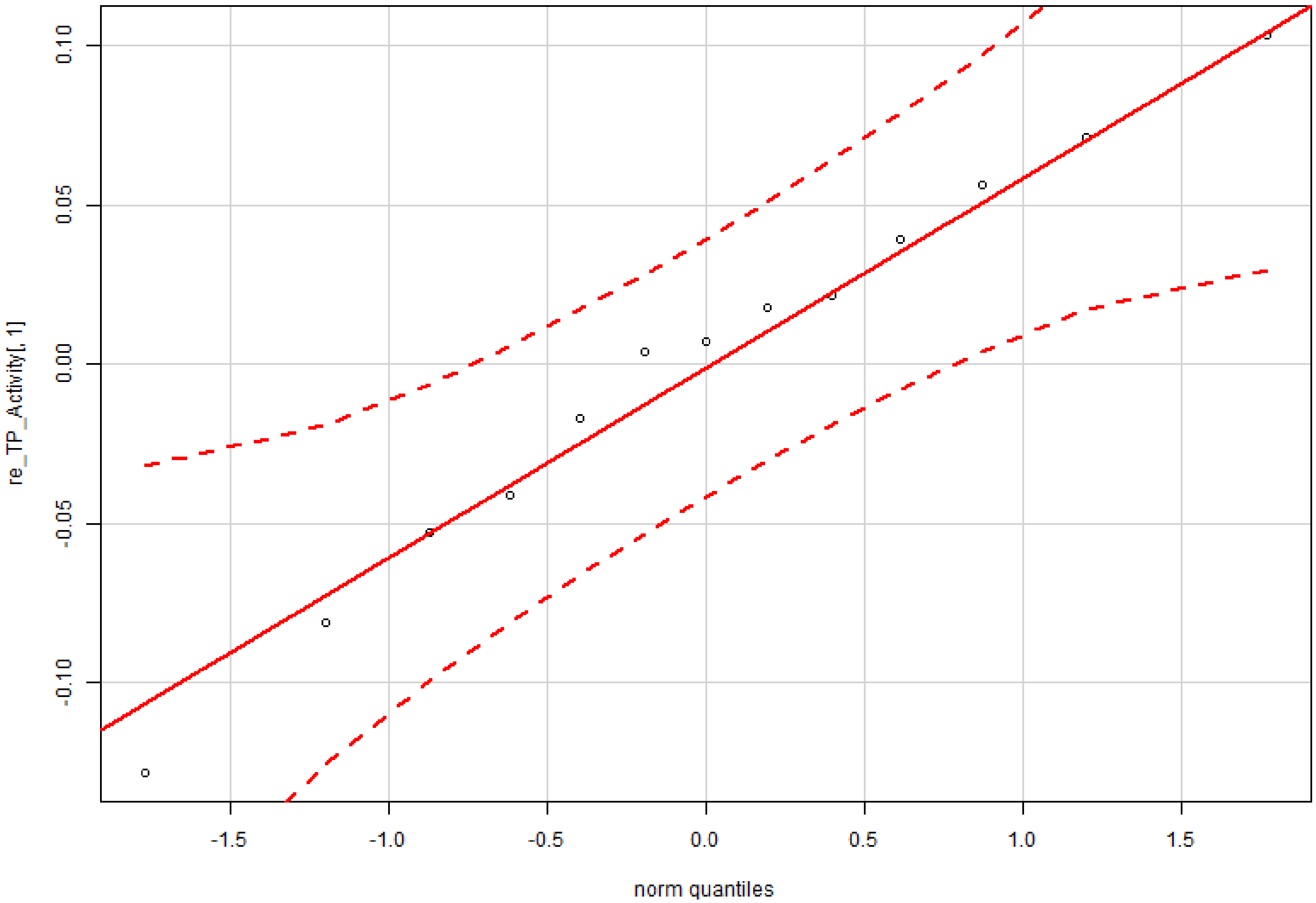

Supplementary Figure S8. Quantile-quantile plot for each level of the random effect - Module A

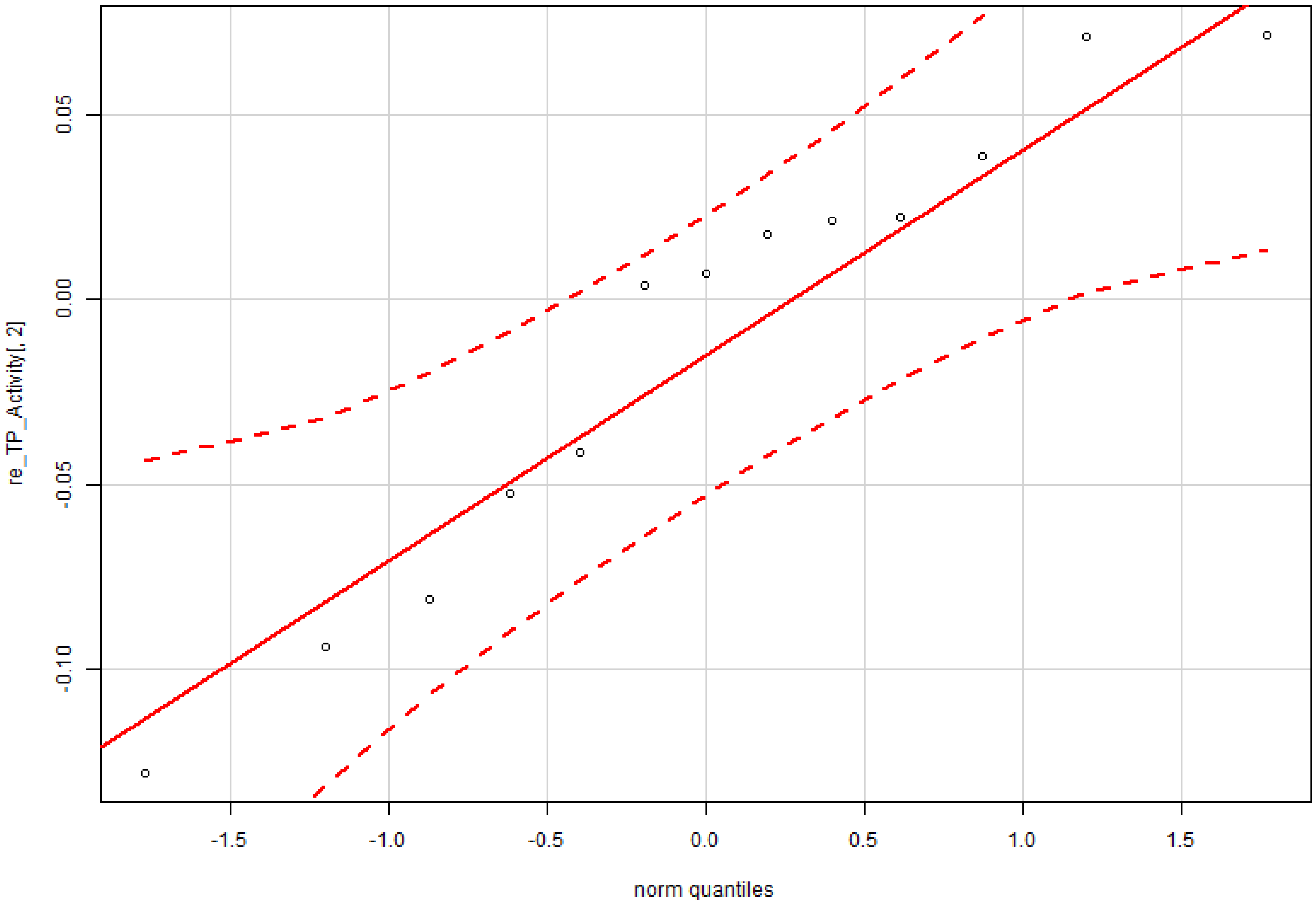

Supplementary Figure S9. Quantile-quantile plot for each level of the random effect - Module B

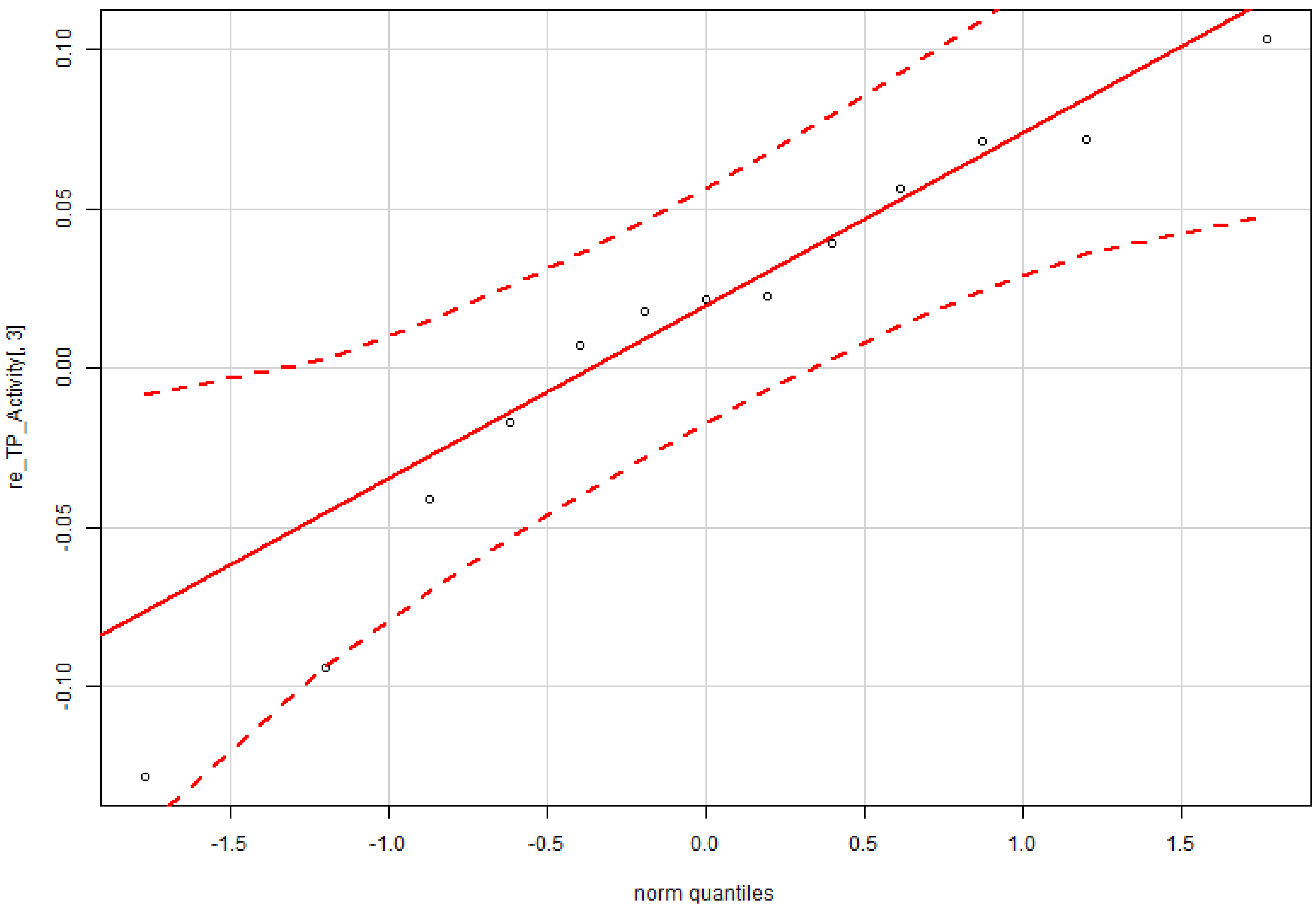

Supplementary Figure S10. Quantile-quantile plot for each level of the random effect - Module C
